# Intrathecal trastuzumab: immunotherapy improves the prognosis of leptomeningeal metastases in HER-2+ breast cancer patient

**DOI:** 10.1186/s40425-015-0084-y

**Published:** 2015-09-15

**Authors:** Nu T. Lu, Jeffrey Raizer, Erwin P. Gabor, Natalie M. Liu, James Q. Vu, Dennis J. Slamon, John L. Barstis

**Affiliations:** Division of Hematology/Oncology, UCLA School of Medicine, 11-934 Factor Building, Los Angeles, 90025 CA USA; Department of Pathology & Laboratory Medicine, UCLA, Los Angeles, 90095 CA USA; Department of Neurology and Division of Hematology and Oncology Northwestern University, Chicago, 60611 IL USA; Jonsson Comprehensive Cancer Center, UCLA, Los Angeles, 90095 CA USA; David Geffen School of Medicine, UCLA, Los Angeles, 90095 CA USA

**Keywords:** Antibody mediated immunotherapy, Intrathecal trastuzumab, HER-2+ leptomeningeal CNS metastases

## Abstract

We describe the clinical and therapeutic course of a 51-year-old woman with HER-2+ breast cancer who developed leptomeningeal (LM) and spinal cord metastases after 8 years of stable disease on combination therapy with intravenous (IV) trastuzumab. Due to progressive CNS disease, intrathecal (IT) trastuzumab was introduced to enhance HER-2+ therapy into the CSF space. A combination HER-2+ targeted approach achieved clinical remission with stable disease in our patient 46 months after she was diagnosed with LM metastases. However, spinal cord C-1 metastasis was not fully controlled with IT trastuzumab, ultimately leading to the patient’s respiratory compromise. In our patient, IT trastuzumab immunotherapy improved prognosis and was an effective strategy to manage HER-2+ LM disease. Given alone or alongside other anti-HER-2+ therapeutics with sufficient CNS penetration, IT trastuzumab could extend the lifespan of patients with leptomeningeal and CNS metastases.

## Background

Trastuzumab (Herceptin; Roche, Basel, Switzerland) is a humanized monoclonal IgG1 antibody which has revolutionized the outcome of patients with HER-2 overexpressing breast cancer. Traditionally, HER-2 overexpressing tumors have been associated with more aggressive disease and inferior prognosis, resulting in shorter survival [1, 2]. However, trastuzumab has transformed HER-2+ breast cancer into one of the most treatable types of cancer. The efficacy of trastuzumab partially relies on its immunologic ability to recruit natural killer lymphocytes to HER-2+ tumors via the antibody dependent cellular cytotoxicity (ADCC) mechanism [3].

Trastuzumab’s control of systemic disease in HER-2+ breast cancer patients has led to a higher incidence of CNS metastases [4]. HER-2+ metastases can develop in areas where intravenous (IV) trastuzumab has little to no penetration, particularly the central nervous system (CNS) where brain and leptomeningeal (LM) metastases can occur. The development of this complication warrants a multi-faceted approach. Extramedullary leptomeningeal metastases require drug penetration through the blood–brain barrier or blood CSF barrier. Intramedullary metastases (brain and spinal cord) bury deeper in the neural tissue and are therefore more difficult lesions to treat. As trastuzumab is highly effective against systemic HER-2+ breast cancer, a logical strategy is to investigate its ability to target HER-2+ CNS metastases.

As a single-agent first line therapy for HER-2+ breast cancer, trastuzumab achieved a response rate of 25 –35 % [5]. Among women with metastatic breast cancer who progressed after standard chemotherapy, trastuzumab yielded response rates of 15–18 % [6] as a second and third line therapy. Several adjuvant (NSABP B-31, BCIRG 006, NCCTG N9831, HERA) [7–9] and neoadjuvant (NOAH, Neo-ALTTO) [10, 11] trials have demonstrated that combining trastuzumab with standard chemotherapy reduces the risk of recurrence and mortality compared to chemotherapy alone in these patients.

Trastuzumab benefits women at any stage of HER-2+ breast cancer and is currently part of the standard of care. Given its demonstrated safety and efficacy, using trastuzumab to target HER-2+ tumor cells that have spread to the cerebrospinal fluid (CSF) by intrathecal administration is a plausible strategic intervention for impacting the prognosis for patients with LM metastases. Yet, intrathecal (IT) trastuzumab has not been formally tested in a prospective fashion. Despite accumulating anecdotal reports suggesting tolerability and good antitumor activity for leptomeningeal metastases [12–16], IT trastuzumab is underutilized as a treatment modality for HER-2+ LM from breast cancer. Our challenge remains: To implement an effective dose schedule of IT trastuzumab in combination with other standard therapies to manage both the extramedullary and intramedullary HER-2+ CNS lesions.

We report a case of a patient who developed LM disease after achieving clinical remission of her metastatic HER-2+ systemic disease with IV trastuzumab. By initiating IT trastuzumab in combination with modalities that can deliver anti-HER-2 therapy across the blood–brain barrier, we halted the progression of HER-2 invasion in the CSF and achieved the longest control of LM disease ever reported to date (>46 months).

## Case Presentation

The patient’s timeline of disease progression and treatment is outlined in Table 1. In April 2003, a 41-year-old woman was diagnosed with stage II breast cancer (T1N1M0, ER/PR-, HER-2/ 3+ by immunohistochemistry). She was treated with surgery and adjuvant chemotherapy (Adriamycin/Cytoxan, and weekly docetaxel). In March 2007, she developed liver, lungs, lymph nodes, and skeletal metastases. She received gemcitabine plus docetaxel, concurrent with IV trastuzumab and zoledronic acid. Subsequent scans showed significant response with clinical remission.

**Table 1:** Treatment Timeline

Date	Disease Progression	Treatment	Serum CA 27.29 (U/mL)
April-2003	Diagnosed with stage II breast cancer	Surgery and adjuvant chemotherapy (adriamysin/cytoxan, and weekly docataxel)	na
March-2007	Developed liver, lungs, lymph nodes, and skeletal metastases	Chemotherapy (gemcitabine/docetaxel with concurrent IV trastuzumab/zoledronic acid), resulted in clinical remission	na
April-2010	Loss of hearing and left facial with recurrence in left internal auditory canal and LM	Cyberknife therapy for IAC tumors and chemotherapy (lapatinib/capecitabine), resulted in significant improvement in neurological symptoms and imaging	na
October-2010	Ommaya inserted	Experimental IT trastuzumab (5 mg flat dose per week)	12.3
January-2011	New parenchymal, thoracic and lumbar LM metastases with additional 5th cerebral nerve and c1 lesions	Whole brain and spine radiation and increased IT trastuzumab (10 mg per week)	12.6
July-2011	Worsening intramedullary c1 lesions	One month of IT trastuzumab (50–80 mg per week) and maximal dose of cyber knife therapy	32.8
March-2013	Developed weakness in left hip/leg and enhancement in previously stable thoracic and lumbar lesions	Increased total dose of IT trastuzumab (50–80 mg BIW, dose divided twice per week) administered by lumbar puncture and ommaya reservoir injections	40
May-2013	No discernable change in disease progression	Trastuzumab (administered bimonthly for maintenance at 50 % of the weekly dose) and lapatinib (750 mg, BID five days weekly), resulted in decreased enhancement and activity of thoracic and lumbar lesions	59
June-2013	Diagnosed with meningitis	Extended antibiotic treatment for two plus months (vancomycin)	58
July-2013	Hiatus from IT with an increase in CA27.29	T-DM1	37
August-2013	Removed ommaya		50
September-2013	New ommaya inserted	Restarted IT trastuzumab (40 mg per week)	50
October-2013	Worsening neurological symptoms	IT trastuzumab 50 mg (100 mg per week)	40
August-2014	Patient expired		40

*Na* not available (patient at different clinic)

Three years later, the patient presented with loss of hearing in her left ear and a left facial droop. This led to the diagnosis of recurrence in the left internal auditory canal (IAC) and LM. CSF analysis showed no tumor cells. Cyberknife therapy for IAC tumors as well as treatment with lapatinib (Tykerb; Glaxo-SmithKline, Philadelphia, PA) + capecitabine (Xeloda; Roche, Basel, Switzerland) resulted in significant improvement in neurological symptoms and imaging studies. She declined whole brain radiation and IT methotrexate. She gave informed consent, however, to have an ommaya reservoir placed to begin experimental IT trastuzumab. This study was in accordance with the World Medical Association (WMA) Declaration of Helsinki. Ethical approval was waived by the University of California Los Angeles Office of the Human Research Protection Program (UCLA-OHRPP).

Given the wide range of IT trastuzumab dosages reported in the literature (flat dosing, ranging from 5 to 100 mg weekly), we started with the lowest dose of 5 mg weekly to assess safety and any efficacy. Three months later, new brain parenchymal, thoracic and lumbar spinal LM metastases, plus 5th cerebral nerve and C1 lesions prompted treatment with craniospinal radiation, followed by a higher dose of IT trastuzumab. We first increased the dose to 10 mg, and then later to 25 mg, given every 2 weeks. These measures stabilized known CNS lesions. Six months later, however, a worsening intramedullary C1 lesion suggested that the IT trastuzumab dosage might be inadequate to treat lesions inside of the spinal cord. To treat worsening lesions, IT trastuzumab was increased to 50–80 mg administered once per week for a month. The patient’s C1 lesion also underwent a maximal dose of cyberknife therapy.

The weekly dose escalation of IT trastuzumab resulted in stable disease until March 2013, when the patient developed weakness in her left hip and leg. Magnetic resonance imaging (MRI) revealed enhancement in the previously stable thoracic and lumbar LM lesions. The patient was given an increased total dose of IT trastuzumab, administered twice weekly by lumbar puncture and ommaya reservoir injections (50–80 mg divided twice per week × one month). After one month, the dose was tapered down and administered every 2 weeks, for maintenance at 50 % of the weekly dose. To further target intramedullary lesion, we implemented a pulsing oral lapatinib regimen (750 mg) twice a day (BID) for five days out of seven days until clinical or radiological evidence of tumor progression [17]. These treatments resulted in decreased enhancement and activity of the thoracic and lumbar lesions on the spinal cord.

The increase in dosing and frequency of IT trastuzumab was generally well tolerated, except for an episode of meningitis in June 2013, which required removal of the ommaya reservoir and over 2 months of antibiotic treatment. After completion of antibiotics, a new ommaya reservoir was inserted. Without IT trastuzumab, a significant increase in the serum CA27.29 tumor marker was successfully normalized with a short course of T-DM1 administered in July 2013 treatment.

**Fig. 1:**
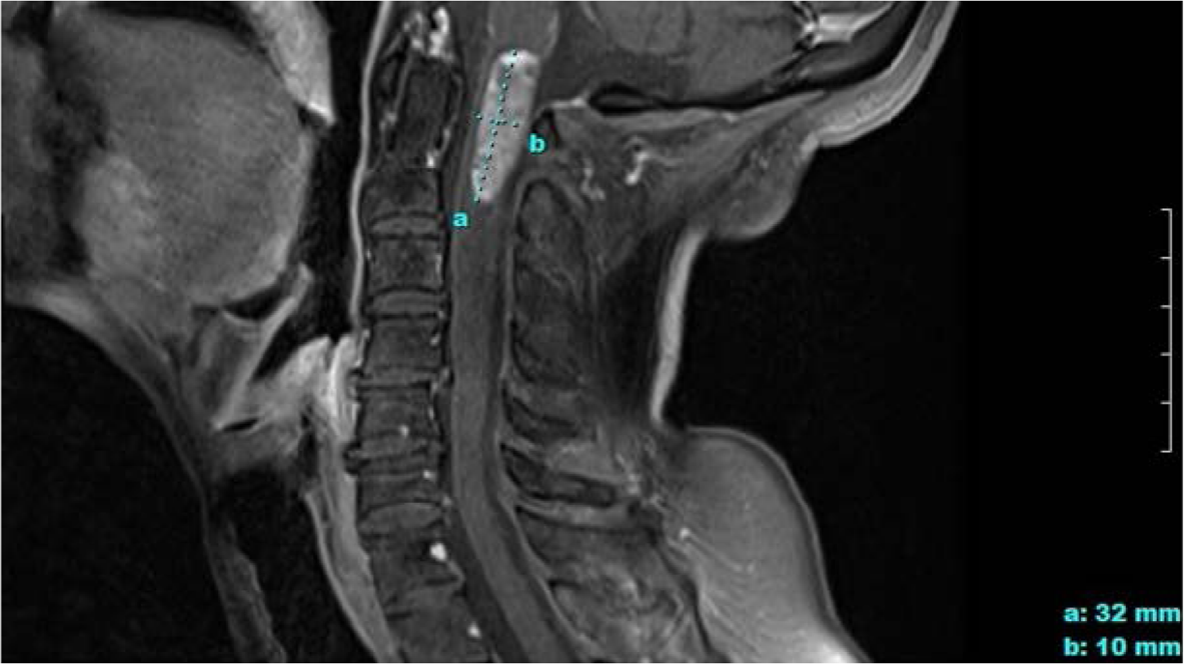
Intramedullary lesion at C1-C2 with associated cord edema

Three and a half years after having started IT trastuzumab, our patient experienced further decline in her left sided motor function and was wheelchair bound. The decreasing functional capacity of the left side likely correlated with the worsening C1 intramedullary lesion seen on MRI (Fig. 1). We attempted, once again, a higher dose and frequency of IT trastuzumab, as well as re-evaluating for potentially re-treatable cyberknife lesions without success. Despite physical decline, the patient’s performance status remained sufficient enough to allow her continued parenting of two adolescent children. Eleven years after her HER-2+ breast cancer was diagnosed, she expired from respiratory compromise due to progressive C1 intramedullary lesion.

## Discussion

Pervasive HER-2+ breast cancer cells can travel long distances and break virtually any protective barrier in the body. To combat HER-2+ CNS metastases and improve patient survival, one must have the means to deliver effective anti-HER-2 immunotherapy to any site of disease. In order to improve prognosis, there is a need for a multi-modality approach against HER-2+ cells in the CSF that create LM, brain, and spinal cord lesions.

IT trastuzumab was effective in targeting extramedullary (LM) metastases. This resulted in the prolonged survival of our patient for 46 months, compared to a median life expectancy of 3–4 months for breast cancer patients following the diagnosis of LM without treatment [18]. Given that IV trastuzumab does not cross the blood–brain barrier or blood CSF barrier, IT trastuzumab offers a direct approach to the leptomeninges. IT trastuzumab immunotherapy delivers an optimal drug concentration to the CSF and is surprisingly well tolerated compared to chemotherapy, such as IT methotrexate. IT methotrexate can be associated with severe side effects including meningoencephalopathy, leukoencephalopathy, stomatitis, and marrow suppression [19].

Furthermore, dose escalation of IT trastuzumab enabled disease management in our patient. When imaging revealed a worsening of old lesions, the appearance of new spots, or when new neurological symptoms emerged, increasing the dose of trastuzumab achieved subsequent stable scans and slowed the patient’s functional decline.

High-dose IT trastuzumab, however, must be administered with great caution. In our experience, CSF proteins that may cause cerebral edema and/or intracranial hypertension can increase with doses higher than 50 mg weekly (Table 2). To prevent this, we have administered up to 80 mg IT trastuzumab weekly in divided doses (40 mg biweekly × 4 weeks) (Table 3). These higher doses were given concurrently via lumbar puncture and ommaya reservoir to ensure more uniform drug distribution in the CSF.

**Table 2:** CSF Analysis on Weekly 50 mg IT Trastuzumab

Week	1	2	3	4
Protein (g/L)	31	52^a^	50^a^	68^a^
RBC (/cmm^3^)	519	74	15	<1
WBC (/cmm^3^)	<1	3	2	<1

**Table 3:** CSF Analysis on Biweekly 40 mg IT Trastuzumab (Total 80 mg Weekly)

Week	1	2		3		4	
Protein (g/L)	34	28	33	26	30	31	61^a^
RBC (/cmm^3^)	<1	1	<1	1	<1	1	1
WBC (/cmm^3^)	<1	<1	<1	1	2	1	<1

CSF samples were obtained prior to IT Trastuzumab injection

*IT* intrathecal, *CSF* cerebrospinal fluid, *RBC* red blood cell, *WBC* white blood cell

^a^indicates high CSF protein

If new CNS lesions were to appear in the lower spinal cord, a CSF flow study might help to assess ommaya administered drug dristribution. If there is poor flow, we hypothesize that lumbar puncture administration in addition to ommaya injection would allow trastuzumab to reach the lumbar and thoracic LM areas more easily. This could rapidly stabilize lower spinal cord LM lesions and minimize further neurological deterioration.

In our experience, imaging and neurological symptoms have been more sensitive than cytology in monitoring LM/CNS progression. Although one other study has documented that serial CSF cytology has 90 % sensitivity in diagnosing LM/CNS progression [20], we have never had a positive cytology in this case. Despite cytology’s low sensitivity in our patient, we noted her elevated serum CA27.29 correlated with worsening neurological symptoms. Thus, the serum CA27.29 tumor marker has provided an additional way to follow our patient’s LM/CNS progression.

Although IT trastuzumab treatment of extramedullary metastases has greatly extended the patient’s lifespan, intramedullary metastases remain a problem and are responsible for her functional decline and eventual death. In order to address this problem, more effective methods will have to be developed to target intramedullary metastases. Currently, treatments for intramedullary lesions include radiation, stereotactic radiosurgery, chemotherapy, microsurgery, and steroids [18]. These non-immunotherapeutic options can be associated with high neurotoxicity and disease recurrence. Each of these options has limitations. Surgery may not always be an option due to tumor location or disease burden. Tissue tolerance may prevent multiple treatments with radiation as it can cause radiation necrosis. Chemotherapy can have systemic and neuro-toxicity and may not penetrate into the CNS. In addition, the standard non-immunotherapeutic option using IT methotrexate only has a median overall survival of approximately 2–7 months in those with breast cancer following the diagnosis of LM with standard IT chemotherapy [21]. In contrast, IT trastuzumab immunotherapy could improve the prognosis of HER-2+ LM disease, as demonstrated by our patient’s 46 month survival.

A comprehensive, targeted approach against HER-2+ CNS disease is in sight. A phase I/II dose escalation of IT trastuzumab (Intrathecal Trastuzumab for Leptomeningeal Metastases in HER2+ Breast Cancer; ClinicalTrials.gov number, NCT01325207) by Dr. Jeffrey Raizer, is underway. This trial is now in phase II and unpublished data presented by Dr. Jeffrey Raizer's team suggests that there is more activity at the highest doses used (Phase I trial of intrathecal trastuzumab in HER2 positive leptomeningeal metastases, 19th Annual Scientific Meeting and Education Day of the Society for Neuro-Oncology). Based on our experience, we predict that the high efficiency of trastuzumab makes it a promising candidate to favorably impact the prognosis of patients with LM metastases from HER-2+ breast cancer.

While its activity on extramedullary lesions is apparent, IT trastuzumab alone isn’t adequate to prevent brain or intramedullary metastases. Lapatinib, a tyrosine-kinase inhibitor that targets HER-2, together with capecitabine, provide a sound, concomitant treatment that has displayed clinical activity against brain metastases from HER-2 overexpressing breast cancer [23]. Stereotactic radiosurgery can control CNS parenchymal disease progression, if its dose has not been maximally implemented. Moreover, trastuzumab could be combined with other antibody based therapies like pertuzumab (Perjeta; Roche, Basel, Switzerland) to improve response rates mirroring the synergistic mechanisms of new treatments for systemic metastatic HER-2+ disease [24]. However, dual antibody treatment requires consideration when treating the CNS as there can be an issue with higher protein in the CSF.

Other immunotherapeutic advances to improve HER-2+ disease prognosis are also currently in development. Trials now focus on the discovery of tumor-associated antigens (i.e., E75-specific CD8 + T cells that lysed HER-2/neu expressing tumor cells) to create vaccines that further stimulate the patient’s own immune system [25]. Peptide-based vaccines use these antigenic epitopes to induce immune regulators, including: antibodies, helper T-cells, and cytotoxic T-lymphocytes. The immune regulators, then, recognize and lyse tumor cells expressing the surface immunogenic peptide [26]. The concept of using immune cells to carry out anti-tumor activities is also apparent in how T-cell immunotherapy can mediate durable regressions of several refractory solid tumors [27, 28]. Similarly, checkpoint inhibitors are immune-oncologic agents with promising activity on melanoma brain metastases [29]. Preliminary findings suggest that this class of compounds may have an extended role against other CNS metastases. More clinical studies will be needed to improve these new immunotherapeutic interventions when targeting CNS disease in the future.

## Conclusion

This is an exciting era of using immunotherapy for HER-2+ cancers. In particular, trastuzumab (Herceptin) has paved the way for HER-2+ breast cancer patients to fight their disease. Due to the incidence of CNS involvement in these patients, there is a great need for an effective approach to target HER-2+ lesions in the leptomeninges and parenchyma of the spinal cord and brain. A number of case studies provide favorable evidence to support the conclusion that IT trastuzumab extends the lives of women with HER-2+ LM metastases. We recognize its role in improving patient prognosis and supporting innovative IT immunotherapy as a crucial component for the management of HER-2+ LM disease.

## Consent

Written informed consent was obtained from the patient for publication of this case report and any accompanying images. A copy of the written consent is available for review by the Editor-in-Chief of this journal.

## Abbreviations

HER-2: HER-2/neu, Human epidermal growth factor receptor 2
HER-2+/3+: Human epidermal growth factor receptor 2/3 positive
LM: Leptomeningeal metastases
IV: Intravenous
IT: Intrathecal
CNS: Central nervous system
CSF: Cerebral spinal fluid
C-1: Cervival-1
ADCC: Antibody dependent cellular cytotoxicity
IgG1: Immunoglobulin G1
NSABP B-31: National Surgical Adjuvant Breast and Bowel Project Protocol 31
BCIRG 006: Breast Cancer International Research Group Study 006
NCCTG N9831: North Central Cancer Treatment Group Study N9831
HERA: Herceptin Adjuvant Trial
NOAH: Neoadjuvant Herceptin Study
Neo-ALTTO: Neoadjuvant Lapatinib and/or Trastuzumab Treatment Optimisation Study
T cell: T lymphocytes
T1N1M0: Tumor node metastasis staging system
ER/PR-: Estrogen receptor/progesterone receptor negative
IAC: Internal auditory canal
MRI: Magnetic resonance imaging
BID: Twice a day
CA27.29: Cancer antigen 27.29
T-DM1: Trastuzumab emtansine
CD8+: Cluster of differentiation 8+
RBC: Red blood cell
WBC: White blood cell
Mg: Milligram
g/L: Gram per liter
/cmm^3^: Per cubic millimeter cubed = microliter
